# Editorial: Endocrine disrupting chemicals in reproductive health, fertility, and early development

**DOI:** 10.3389/fendo.2024.1478655

**Published:** 2024-08-30

**Authors:** Maria De Falco, Laura A. Favetta, Rosaria Meccariello, Kristina Pogrmic-Majkic, Terje Svingen

**Affiliations:** ^1^ Department of Biology, University of Naples Federico II, Naples, Italy; ^2^ National Institute of Biostructures and Biosystems (INBB), Rome, Italy; ^3^ Reproductive and Health Biotechnology Lab, Department of Biomedical Sciences, Ontario Veterinary College, University of Guelph, Guelph, ON, Canada; ^4^ Department of Medical, Human Movement and Well-being Sciences, University of Naples Parthenope, Naples, Italy; ^5^ Department of Biology and Ecology, Faculty of Science, University of Novi Sad, Novi Sad, Serbia; ^6^ National Food Institute, Technical University of Denmark, Lyngby, Denmark

**Keywords:** endocrine disruptors, reproduction, reproductive health, fertility, mode of action

The rising incidence of reproductive disorders in both men and women over the past few decades presents a significant global challenge, impacting societies and individuals worldwide. Environmental chemical exposure, including endocrine disrupting chemicals (EDCs), is recognized as a contributing factor to this increasing disease burden (1).

EDCs are known to disrupt sexual development by interfering with androgen, estrogen, and steroidogenesis pathways (2). However, there are still numerous unanswered questions regarding how these disruptions qualitatively and quantitatively lead to various diseases. Additionally, our understanding of EDCs’ impacts through alternative mechanisms and signaling pathways is limited. Gaining this knowledge is crucial for ensuring human health, especially as regulatory toxicology increasingly moves away from animal testing towards alternative test methods for hazard identification and risk assessment.

This Research Topic was launched to advance our understanding of how EDCs disrupt reproductive development and contribute to disease. Despite extensive research over the past three decades, many fundamental mechanisms of EDC effects in complex organisms, including humans, remain elusive. As regulatory toxicology shifts towards greater reliance on non-animal testing, it is essential to develop a clear mechanistic understanding to inform the creation of robust alternative testing strategies capable of identifying harmful chemicals for regulatory purposes or further *in vivo* testing.

The first paper in this Research Topic, by Motawee et al., explores the potential ameliorating effects of ginger on cadmium-induced testicular toxicity in adult rats. Cadmium is a naturally occurring metal found in the Earth’s crust and used in various industrial applications, including battery production, pigments, coatings, plastics, and cigarettes (3). It is toxic to humans and animals and can affect many organ systems such as kidneys, bones and the reproductive system when exposure occurs through ingestion, inhalation, or environmental contamination (4). In their study, Motawee et al. observed significant testicular toxicity following four weeks of exposure to 4 mg/kg/day of cadmium chloride (CdCl_2_), including reduced testis weight and testis dysgenesis (e.g. disrupted seminiferous tubules) alongside changes in systemic steroid hormone and gonadotropin levels. However, daily co-administration of high levels of ginger (4 mg/kg/day) protected against many of the toxic effects, with preserved tissue architecture and improved systemic hormone levels. Although the exact mechanisms underlying the ameliorating effects unclear, the authors propose free-radical scavenging, anti-inflammatory, antioxidant and anti-lipid peroxidase effects.

The second paper by Kourmaeva et al. shows how bisphenols A and F (BPA, BPF) can induce apoptosis in granulosa cells of cow ovaries via the intrinsic mitochondrial pathway. Bisphenols, particularly BPA, have for long been known to possess endocrine disrupting properties potentially affecting numerous biological processes and organs, including female fertility (5). With a worrying decline in fertility rates across the globe (6) and with EDCs hypothesized to contribute to decline in female reproductive health (7), the continued study into both mechanisms and adverse outcomes of exposure to environmental chemicals is important. In this study, both BPA and BPF affected granulosa cell morphology and confluency, but interestingly these effects were not observed for a third tested bisphenol, BPS. Through molecular analyses, the apoptotic pathway was disrupted in the granulosa cells, pointing towards a mechanism in which some bisphenol analogues can negatively impact ovarian health by inducing granulosa cell apoptosis, ultimately affecting the follicles necessary for female reproductive health and fertility.

The third paper by Corpuz-Hilsabeck and Culty provides a synthesis of current literature about how environmental chemicals and pharmaceuticals with endocrine disrupting potentials can affect the testicular Sertoli cells, ultimately affecting male reproductive development and function. Sertoli cells represent a key somatic cell type of the testis that is central for early gonad differentiation, then as nursing cells for the germ cells supporting the central testis function of sperm production (8, 9). Regarding male reproductive toxicity and EDCs, however, much focus has been on testicular Leydig cells and steroid sex hormones, with less focus looking into direct effects on the supporting Sertoli cells. This review highlights an existing knowledge gap regarding mechanisms by which environmental chemicals and pharmaceuticals can affect the Sertoli cells and subsequently contribute to various health effects. This synthesis clearly shows how many substances, from bisphenols and phthalates to genistein and paracetamol, can disrupt Sertoli cell integrity or function, but also shows a lack of studies looking into effects of chemical mixtures on this central cell lineage.

The fourth paper by Guo et al. examines potential association between use of personal care products (PCPs) and reproductive outcomes in *in vitro* fertilization/intracytoplasmic sperm injection (IVF/ICSI) treatment. Personal care products encompass a wide array of consumer goods used for hygiene, beautification, and grooming, including items such as soaps, lotions, deodorants, cosmetics, toothpaste, and sunscreens. These products often contain a variety of chemical ingredients designed to cleanse, protect, and enhance the appearance and health of skin, hair, teeth, and other body part; but unfortunately, many of these ingredients may also possess negative properties such as endocrine disrupting potentials. Common EDCs found in personal care products are BPA, phthalates and parabens, which all may affect human health, including reproductive development and fertility (10). This cohort study includes 1500 women seeking ART treatment at the Reproductive Medicine Center of Tongji Hospital, China, and evaluates use of personal care products as reported by questionnaires relative to reproductive outcomes of IVF/ICSI procedures. The study reports that women more frequently using skin care products had lower oocyte maturation rates than those who did not use skin care products, and women using cosmetics had a higher risk of miscarriage after transfer of fresh embryos. This study thus provides information on the associations between PCPs and adverse reproductive outcomes, but the authors stress the need for further epidemiologic studies to validate their findings.

This small collection of papers again demonstrates how various environmental chemicals and pharmaceuticals can exhibit endocrine-disrupting activities, leading to disruptions in reproductive development and function. Despite being only four studies, they underscore the complex nature of EDCs and their interactions with biological systems. While traditional EDC assessments have predominantly focused on the EATS modalities (estrogenic, androgenic, thyroid, and steroidogenesis pathways), it is increasingly evident that many other mechanisms may contribute to adverse health outcomes caused by endocrine disruption. These outcomes often hinge on how endocrine disruption is defined and understood. Future research is essential to elucidate these additional pathways, with the hope that this growing body of knowledge will be instrumental in enhancing our ability to safeguard human health from the harmful effects of chemical exposures.
